# Analysis of Failure Mechanics in Hybrid Fibre-Reinforced High-Performance Concrete Deep Beams with and without Openings

**DOI:** 10.3390/ma12010101

**Published:** 2018-12-29

**Authors:** Piotr Smarzewski

**Affiliations:** Department of Structural Engineering, Faculty of Civil Engineering and Architecture, Lublin University of Technology, 20-618 Lublin, Poland; p.smarzewski@pollub.pl; Tel.: +48-81-538-4394

**Keywords:** fibre-reinforced high-performance concrete, deep beams, steel reinforcement, steel fibres, polypropylene fibres

## Abstract

The article presents the results of experimental- and analytical investigations of the behaviour and the load-carrying capacity of deep beams with openings (DBO) and without openings (DB) made of hybrid steel-polypropylene fibre-reinforced high-performance concrete (HFRHPC) subjected to three-point bending tests. Six deep beams 100 mm × 500 mm × 1000 mm were tested with a gradually increasing load until failure. All the specimens were tested in the same simply supported conditions. The research focused on the quantity and kind of concrete reinforcement. The deep beams with steel and polypropylene (PP) fibres were characterised by variously arranged steel bar reinforcement: vertically, horizontally, orthogonally and diagonally. The DB1, DBO1 deep beams were conventionally made with steel rod reinforcement but without fibres. The steel wire mesh reinforcement was replaced by fibre reinforcement of varying volume percentages in the remaining deep beams. The influence of the hybrid fibre content in the specimens was studied by marking the development and propagation of cracks, by recording the failure modes, and by monitoring the deflections at the bottom of the deep beam, at the mid-span and at the support. Three-dimensional measurements of strain and displacement of the deep beams without openings (DB) were performed by the non-contact optical 3D deformation measuring system ARAMIS. The experimental results were compared with the studied methods of predicting the shear strength of deep beams reinforced with hybrid fibre. The conducted study demonstrates that hybrid fibres as web reinforcement have a favourable impact on deep beam crack widths and raise the load carrying capacity of deep beams with openings.

## 1. Introduction

The key weakness of high-performance concrete (HPC) is its brittleness. Numerous researchers have revealed that different types of fibres can be used to remedying the brittle nature of HPC as well as help improve tensile strength and ductility [1,2,3,4,5].

The methods of obtaining fibre-reinforced high-performance concrete consist in modifying ordinary fibre reinforced concrete by appropriate dosage of carefully selected ingredients and a proper curing process. Fibre-reinforced concrete (FRC) is a quasi-plastic composite material consisting of a brittle concrete matrix and plastic, carbon, glass or organic fibres which perform the function of scattered reinforcement. The role of fibres as reinforcement of composites is discussed in a textbook of Materials Science and Engineering [6]. The presence of fibres in the concrete structure, depending on its type, may reduce cracking development, improve the static and dynamic strength properties, ductility performance, fatigue resistance, bond strength, impact resistance and fracture toughness. These features are desired for traditionally reinforced concrete (RC). Due to the complexity of the FRC structures and the features that distinguish them from RC structures, the use of fibre in concrete is usually based on experimental test results as well as an engineer’s intuition. However, the guidelines for designing FRC have already been standardised in numerous countries [7,8,9,10]. In works [11,12,13,14,15,16,17,18] the characteristics of these composites were presented and the directions for future development were outlined. Another interesting trend observed in recent decades is the use of industrial wastes in concrete in order to achieve satisfactory environmental and public benefits [19]. 

Fibre hybridisation in concrete, i.e. the optimal combination of various types of fibres with different properties in order to develop modern composites characterised by high strength in a wide range of crack openings, has recently drawn a great deal of attention [20,21,22,23,24]. The most ideal combination is long steel fibres that ensure concrete resistance to significant macro-cracks, in addition to short polypropylene (PP) fibres to strengthen the mortar phase, not only to prevent micro-crack initiation but also to hinder macro-crack propagation. The combination of long steel and short polypropylene fibres can significantly improve the flexural toughness, crack control properties, impact resistance and fire resistance of HFRHPC [25].

According to ACI 318-08 [26], a deep beam is defined as a structural member in which the shear span to effective depth ratio is limited to two and the clear span to depth ratio is restricted to four. Deep beams are construction elements used in civil engineering, mostly in high buildings and maritime structures. In many cases it is necessary to making openings in these elements for doors, windows, ventilation ducts and air conditioning ducts. Since the 1970s, numerous experimental and analytical works aimed at testing the behaviour of deep beams have been conducted. The research concerned the load-bearing capacity of these elements, depending on their size, load scheme, support method, distribution, shape and size of openings, web reinforcement configuration, and the method of concreting [27,28,29,30,31,32,33,34,35,36].

Steel fibres in RC structures are used to reduce steel reinforcement [37]. Reducing or eliminating the shear reinforcement in RC structures by fibre application can potentially decrease the costs of the construction as well as the steel rebars congestion [38]. Very little research that deals with deep beam elements reinforced with steel or polypropylene fibres may be encountered in literature [38,39,40,41]. Hybrid fibre reinforcement in concrete can be very advantageous in deep beams with openings, in which the shear capacity determines their performance. No investigation is currently available in the literature on the shear capacity of high-performance concrete deep beams with additions of steel and polypropylene fibres.

The present research concerns concrete deep beams (DB) and concrete deep beams with two symmetrically located openings (DBO) at the middle of the shear span. The research presented in the article was undertaken to determine the influence of the volume content of steel and polypropylene fibres on deep beam cracking and deformation. All the beams were investigated until failure in the three-point flexural test. Analysis of the test results allowed the deep beam shear strength to be evaluated, depending on the steel and polypropylene fibre contents. The impact of deep beam fibre content was investigated by means of measuring the deflections, the strains and the propagation of cracks. The values calculated using analytical formulas [30,33,40] were compared with the shear strength obtained from the experiments. The numerical analysis of the elasticity problem for steel-polypropylene fibres reinforced concrete deep beams considered in this study was described in by Kolesov et al. [42].

## 2. Experimental Procedure/Process

### 2.1. Deep Beam Testing

**Figure 1:**
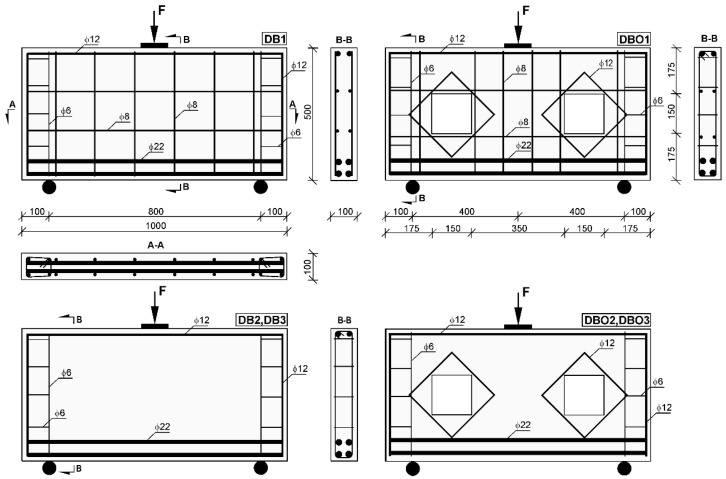
Dimensions, reinforcement arrangement and openings regimes of test deep beams (units in mm).

**Figure 2:**
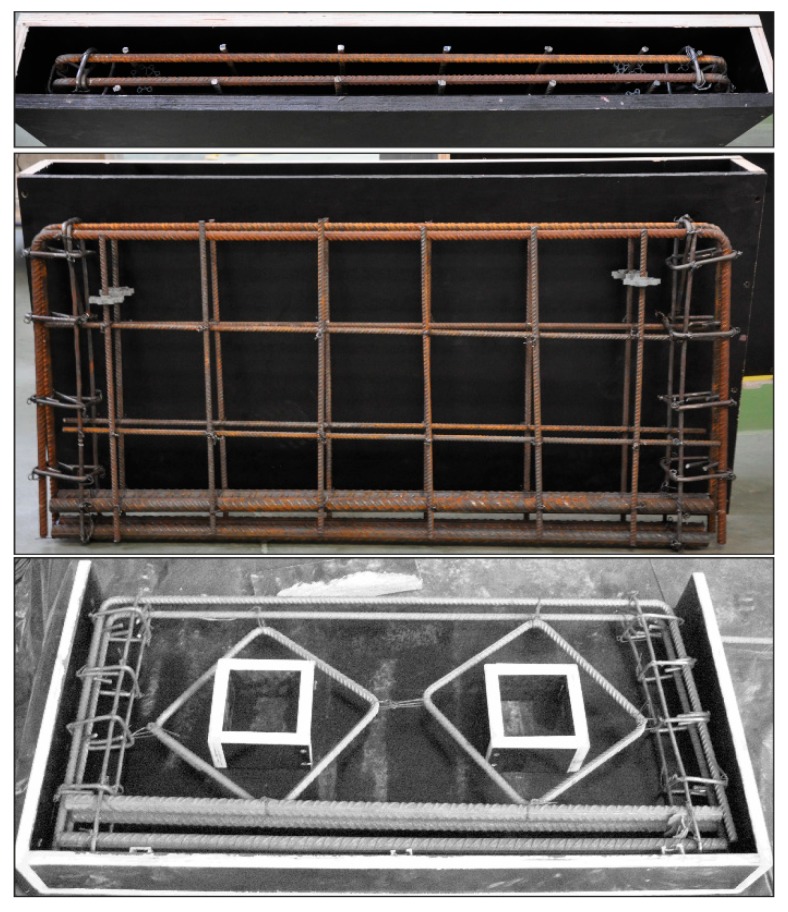
Fabrication of internal steel bars for deep beams DB1, and deep beams with two symmetrically located openings DBO2, DBO3.

Six concrete deep beams were investigated in the research. Each test specimen was 1000 mm in length and a 100 mm × 500 mm rectangular cross section, with different fibre contents. The deep beam had an effective span of 800 mm, with a span-to-depth ratio of 1.6. Every deep beam during testing was subjected to an 0.8 shear span-to-depth ratio. Four Φ22 mm deformed steel bars comprised the tension steel reinforcement, while two Φ12 mm deformed steel bars formed the compression steel reinforcement. Each end of the compression steel reinforcement bars finished with a 90° hook. The web meshes of the DB1 and DBO1 deep beams were made up of Φ8 mm deformed bars. Longitudinal bars located at both sides of the deep beam formed the horizontal web reinforcement, whereas the vertical web reinforcement had the form of stirrups. There were two square openings in each DBO deep beams shear span. In accordance with the 0.3 opening height-to-depth ratio, each opening was 150 mm × 150 mm in size. Two Φ12 mm diagonal steel bars were placed internally around the opening to strengthen the DBO deep beams. Details of the DB and DBO deep beams are presented in Figure 1, while Figure 2 shows a photo of the internal steel bar arrangement.

**Figure 3:**
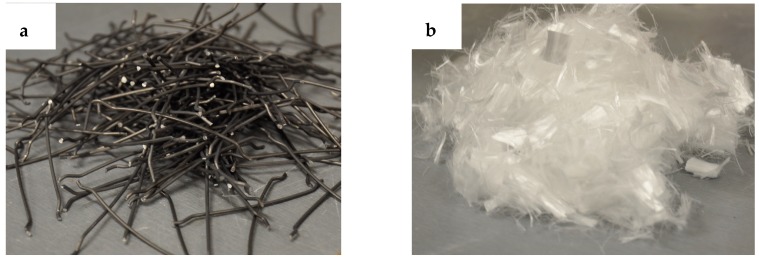
Fibres used in this study (**a**) steel fibres; (**b**) polypropylene fibres.

Four of the deep beams (DB2, DB3, DBO2, DBO3) were made of high-performance concrete with varying steel and polypropylene fibre contents. These fibres replace the web meshes. The hooked-end steel fibres have the following properties: length 50 mm, diameter 1 mm, density 7.8 g/cm^3^, modulus of elasticity 200 GPa and tensile strength 1100 MPa. The hooked ends improve anchoring in the concrete [43,44,45]. The polypropylene fibres were extruded from polypropylene granules, gathered in bundles and cut to 12 mm. They were added to the concrete mixture to decrease the shrinkage and limit cracks in the hardened concrete [46,47]. The polypropylene fibres have the following characteristics: diameter 25 μm, density 0.9 g/cm^3^, modulus of elasticity 3.5 GPa and tensile strength 350 MPa. The polypropylene fibres are smaller in size, whereby they can bridge micro-cracks and control their growth in the HPC. This results in a higher tensile strength of the matrix. By contrast, the steel fibres are larger and they can arrest the development of macro-cracks, and this thereby leads to the improvement in the flexural toughness. For these reasons, the favourable interaction between two different fibres in the hybrid concrete should surpass the interaction of the one type of fibre. Steel and polypropylene fibres are depicted in Figure 3. The DB deep beams had a water/binder ratio (W/B) of 0.19, and for the DBO deep beams it was 0.27. The reduction in the workability of DB concrete was observed due to the large amount of polypropylene fibres tending to lump in balls, and absorb some of the free water required for lubrication and paste formation. Therefore, the water-to-binder ratio (W/B) in deep beams with openings was increased by 42% in order to improve the workability of FRC mixtures compared to DB series and obtain an uniform distribution of the hybrid fibres around the holes. A concrete was made using a highly effective superplasticizer based on polycarboxylate ethers with a density of 1.065 g/cm^3^ at 20 °C so as to achieve the similar workability. Detailed compositions of the concrete mixes used to produce the specimens are given in Table 1.

**Figure 4:**
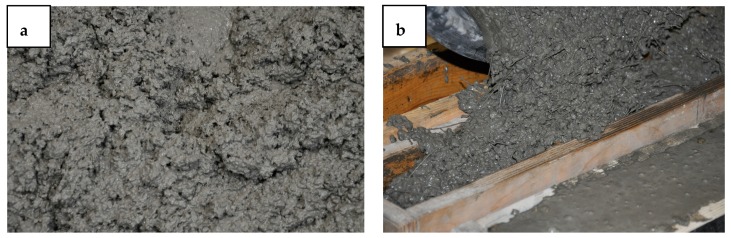
Appearance of fresh mixtures (**a**) high-performance concrete (HPC) and (**b**) hybrid steel-polypropylene fibre-reinforced high-performance concrete (HFRHPC).

Portland cement CEM I 52.5R is characterised by a high initial strength and high heat release in the initial period of curing. Silica fume reduces the porosity and water permeability of concrete, and increases its strength. The aggregate consisted of 0.05/2 mm fraction quartz sand and 2/8 mm fraction granodiorite. Granodiorite is an acidic igneous deep-seated rock with a moderate crystal structure. After drying the aggregate at 105 °C, the quartz sand and granodiorite particle size distributions were ascertained according to the PN-EN 933-1:2012 standard [48]. Superplasticiser causes a significantly higher cement particle dispersion and has the unique ability to provide an appropriate consistency. The use of such ingredients gives a homogeneous concrete mixture with low internal resistance to friction, which significantly improves concrete workability. This is particularly important for fibre-reinforced concrete. Preparation of the mixtures was performed by means of a typical concrete mixer. The mixing commenced by homogenizing the granodiorite and quartz sand with half the quantity of water. Afterwards, the cement and silica fume were added along with the remaining water and at the end the superplasticiser. Once the components were thoroughly mixed, in the DB2, DB3, DBO2, DBO3 mixtures, in order to achieve a homogeneous and workable consistency, the steel and polypropylene fibres were added by hand. Appearances of HPC and HFRHPC mixtures are shown in Figure 4. The deep beams were cast in plywood moulds lying in a horizontal position. In order to maintain the assumed concrete cover, spacers were used. The ready mixtures were placed in moulds and thickened by vibrators. The specimens were covered with foil after compacting in order to minimize moisture loss. Then the specimens were stored for 24 h at a temperature of approximately 23 °C, after which they were removed from the moulds. Finally, the deep beams underwent water bath curing for two weeks. Over the next 14 days, the samples remained in air-dry conditions.

### 2.2. Material Properties

The HPC and hybrid fibre reinforced HPC were subjected to a series of various tests at 28 days of curing to measure the mechanical properties. The tests were carried out on a servo-hydraulic closed-loop testing machine (walter+bai ag Testing Machines, Löhningen, Switzerland) having a 3 MN capacity. Cube-shaped specimens having 150 mm sides were used to test the compressive and splitting tensile strengths, according to the PN-EN 12390-3:2011 [49] and PN-EN 12390-6:2011 [50] standards. Prisms of the dimensions 100 mm × 100 mm × 500 mm underwent 4-point bending tests using a span of 400 mm to determine the flexural tensile properties. The flexural strength was ascertained according to PN-EN 12390-5:2011 [51]. The modulus of elasticity was obtained from Φ150 mm × 300 mm cylinders. A device with an extensometer was employed to carry out static modulus testing in accordance with ASTM C469/C469M-14 [52]. Table 2 presents the concrete mechanical properties.

The addition of steel and polypropylene fibres affect the HPC compressive strength. Compared with the plain HPC, it decreased by 30–35% for DB mixes, and by 0.5% for DBO3 mix. It can be seen that the percentage of polypropylene fibres determined the decrease in compressive strength. At the lowest content of these fibres, the DBO2 mixture strength increased slightly compared to fibreless concrete. Ramakrishnan et al. [53] reported that, FRC with 2% polypropylene fibre volume content had poorer workability, higher entrapped air, and lower unit weight in comparison with control plain concrete, and this resulted in a compressive strength decrease. This observation indicates the importance of adjusting aggregate proportions when high fibres quantities are applied [54]. In addition, the polypropylene fibres create poor void regions in HPC and failure may occur due to them.

By contrast, the hybrid fibres improve tensile strength significantly. Compared with the plain concrete, it increased by 18% to 68%. It was caused by the bridging action of the fibres which transferred tensile stresses after the formation of the cracks, and thus improved the HPC tensile strengths. Mohammadi et al. [55] showed that the addition of 2% hybrid fibre increase splitting tensile strength up to 59% with respect to control concrete. Qian and Stroeven [56] examined the influence of the addition different steel fibres and polypropylene fibre on the flexural strength of concrete at the fibre content up to 0.95%. It was informed that the addition of hooked-end steel fibre and polypropylene fibre significantly increase flexural strength of concrete. Hooked-end steel fibres are the most effective in increasing these strengths. Afroughsabet et al. [57] reported increases in the splitting tensile strength and flexural strength of concrete containing 2% steel fibre up to 143% and 167%, respectively.

Plain steel bars 6 mm in diameter were used for stirrups in accordance with PN-B-03264:2002 [58]. They were fabricated from Class A-I nominally 240 MPa grade drawn wire reinforcement. Deformed, hot rolled steel bars 22 mm, 12 mm and 8 mm were fabricated from Class A-III nominally 410 MPa grade. Axial tensile tests were performed on steel bars with diameters of Φ6, Φ8, Φ12 and Φ22 mm and a length of 300 mm in an MTS 810 hydraulic press with a load range 0–100 kN and the Walter-Bai AG testing machine. The bars were fixed with special holding jaws which prevented potential slide and incorrect measurements. The yield strength, tensile strength, ultimate elongation at maximum force and modulus of elasticity were calculated based on the test results. The mechanical properties of the steel bars are displayed in Table 3.

**Figure 5:**
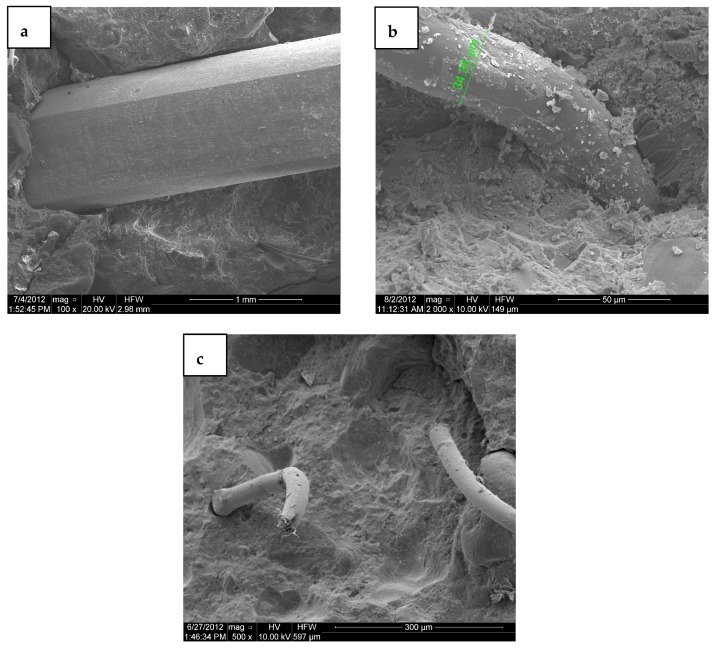
Scanning electron microscope (SEM) images of (**a**) steel fibre; (**b**) polypropylene (PP) fibre in the HFRHPC matrix; (**c**) rupture of PP fibre.

The microstructure of the transition zone of fibres and cementitious matrix has been observed by scanning electron microscope (SEM, FEI Quanta FEG 250, Hillsboro, OR, USA) after mechanical tests. The steel fibre had a smooth surface which was separated from the surrounding matrix, Figure 5a. The steel fibre surface suggests the weaker interfacial bond between steel fibre and concrete matrix which however was compensated by the hooked-ends. The high tensile strength and weaker bond strength perform the steel fibre more receptive to pullout than to rupture, which is an intended effect to allow more ductility. Figure 5b shows that polypropylene fibres are covered by Calcium-Silicate-Hydrate (C-S-H) gel. Their low tensile strength causes them to be rupture, Figure 5c. Thus, the combination of hooked-end steel fibres with high tensile strength and polypropylene fibres with high bond strength can contribute to an overall improvement in the bond strength and more efficient reduction of cracks width in the matrix.

### 2.3. Testing Procedure

**Figure 6:**
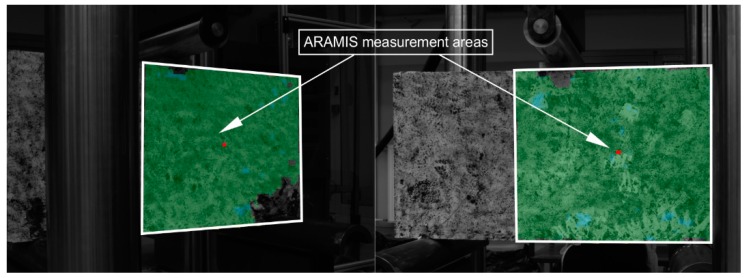
ARAMIS system measurement areas for deep beams (DB).

Before the tests, the deep beams were whitewashed with lime. The surface of the side recorded by two cameras was randomly covered with black paint. Such a procedure is necessary for heterogeneous materials without sufficient characteristic spots for calculations in the ARAMIS system. After setting the deep beam on the stand, this system was calibrated. On the basis of the angle between the cameras’ axes, three-dimensional coordinates were defined from the two-dimensional coordinates from the left and right cameras. The recording speed and measurement area were defined for the calculations (Figure 6).

**Figure 7:**
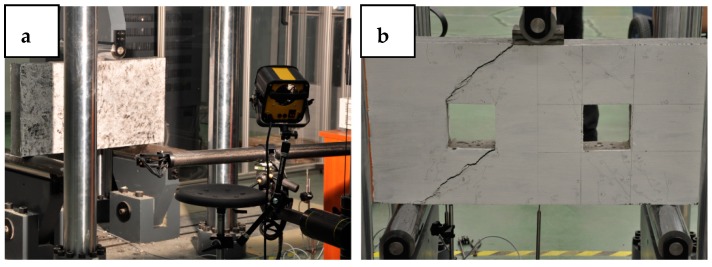
Test station of deep beams: (**a**) without openings DB and (**b**) with openings DBO.

The tests on the deep beams were conducted in a Zwick/Roell hydraulic press with a capacity of 3 MN. Figure 7 shows the test station. In three-point bending with a clear shear span of 350 mm and an effective span of 800 mm, all the deep beams were tested until failure. At the mid-span a steel bearing plate of dimensions 40 mm × 100 mm × 100 mm was used, under the loading point to transfer the load to the specimen. The modes of failure were recorded. Three-dimensional deformation were evaluated in the ARAMIS system in which strains can be measure in a range of 0.01% up to several 100% and the accuracy of displacements measurements is 5 μm [59]. The deformations were computed and analysed for the DB. On the basis of the photos taken by the digital cameras, the ARAMIS system recognised the surface of the measured object (each pixel in the photograph has its own coordinates). After recording the data, comparing the photographs, defining the global coordinates of the system and the interpolation of the missing points, the displacements and strains were calculated. More details about the use of ARAMIS system to measuring the displacements and strains are provided in [25].

The development and propagation of cracks were marked for DBO with openings. Linear variable displacement transducers (LVDT) fixed at the bottom of the deep beam, at the support, and at the mid-span, monitored the deep beam deflections.

## 3. Experimental Results and Discussion

### 3.1. Load-Deflection Curves

**Figure 8:**
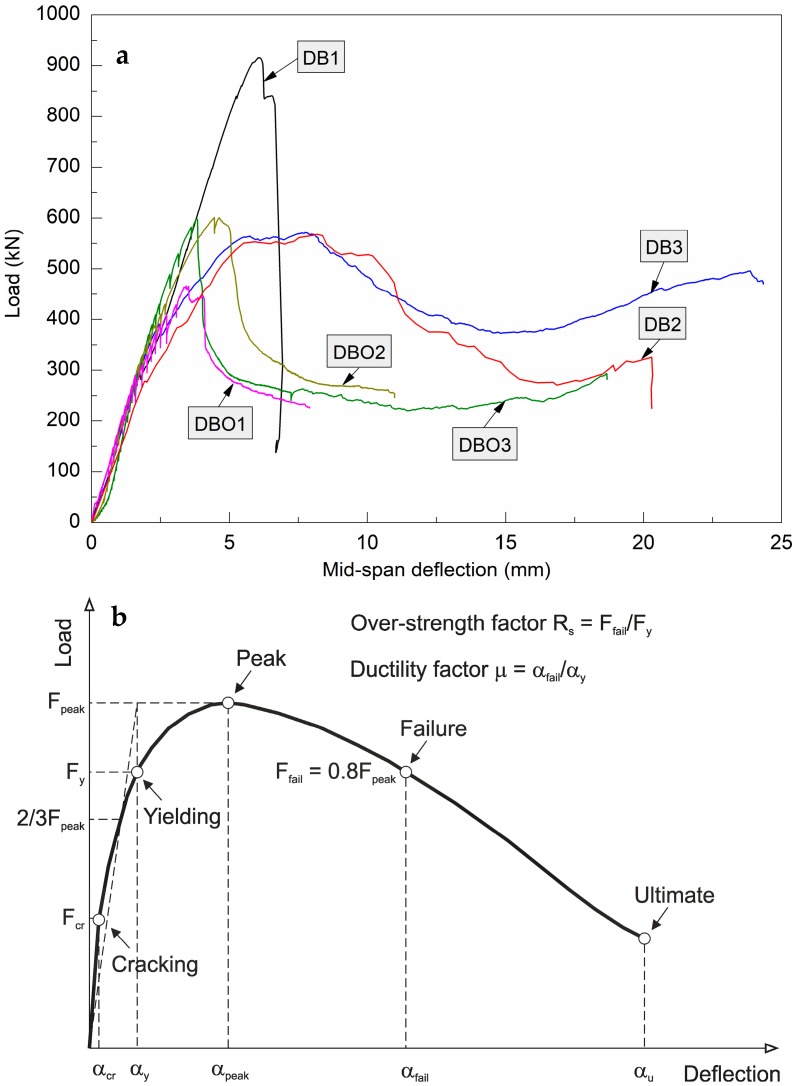
DB and DBO (**a**) load-deflection curves; (**b**) definition of cracking point, yielding point, failure point, over-strength factor and ductility factor.

Figure 8a presents the load-deflection curves for DB and DBO with openings. The fibre content in the deep beams varies in the range 0–2% steel fibre and 0.05–0.5% polypropylene fibre for the six different mix proportions considered in this study. The variations in deflection with load are almost linear until the first macro-crack is appeared. Reduced stiffness of the deep beams at higher deflection rates was seen as more cracks initiated and propagated. Moreover, the fibre volume content had a great effect on the ultimate deflection. An increase in the ultimate deflection was noticed as the fibre volume content was raised. In contrast, the peak load (F_peak_) fell as the fibre volume content was increased. The peak load fall in DB specimens compared to DBO specimens with a lower fibre volume content was due to the lower compressive strength of DB specimens associated with a high PP fibre content and low W/B ratio. Failure of DB occurred as a result of FRC crushing in compressed zones, so compressive strength had a decisive influence on the peak loads obtained. It is true that DBO had a lower fibre content compared to DB elements, but more steel bars were used in them. In general, the total weight of reinforcement used in traditionally RC deep beams was 4.5–9.2 times greater than the total weight of FRC deep beams.

Table 4 presents the load-deflection response results for all the specimens. Figure 8b illustrates definition of the cracking load, yielding load, failure load, over-strength factor and ductility factor. Cracking load (F_cr_) is the point in which the first macro-crack was observed [60]. The yielding load (F_y_) was identified as yielding of tensile reinforcement. The secant stiffness at the point of two thirds of the peak load (2/3F_peak_) was applied to idealize the elastoplastic line which passes through the peak point of the load–deflection curve, and then the deflection at an intersecting point between the two lines was used to determine the yielding load on the curve [61]. The failure load (F_fail_) was calculated as the 80% of the peak load [62].

It can be seen that increasing the fibre volume content of the deep beams in general caused growth in their cracking loads; also for the deep beams with the openings. These increments in cracking loads (36% for DB2; 8–15% for DBO) arises from the corresponding decrease in the W/B ratio of the FRC and a rise in flexural strength. An increase in the yielding loads was detected with a growth in fibre content. The decrease was 97–100% for the DB deep beams as compared to the deep beam without fibres. On the other hand, the increase was 33–36% for the DBO deep beams with the openings. The increase in the yielding load of the deep beams DBO is consequence of higher W/B ratio, and lower total content of the fibres associated with a different arrangement of reinforcement. This demonstrates that the configuration of the steel reinforcement and hybrid fibres used in this study, particularly in deep beams with openings is beneficial. Similar relations were observed in the peak loads. Steel and polypropylene fibres in the amount of 1% and 0.05%, respectively, provided the great benefits. For the deep beams with higher fibre content, a drops in the peak load and changes in the failure mode were observed as will be discussed in the following section.

### 3.2. Cracking Behaviour and Failure Modes

**Figure 9:**
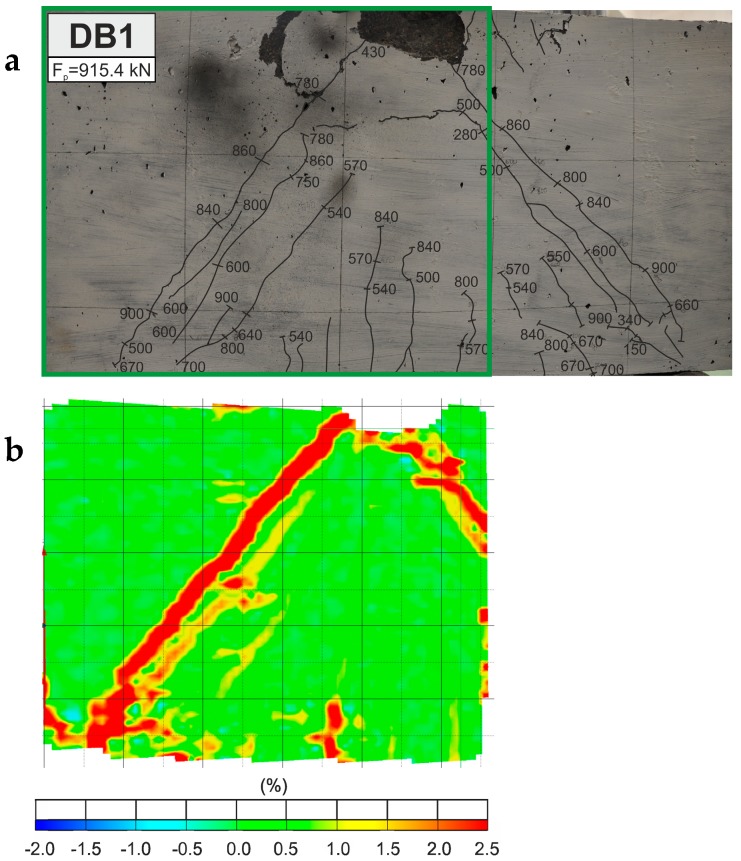
Reinforced concrete deep beam without fibres (on the opposite faces) (**a**) crack propagation; (**b**) contour of principal strains.

During load increase to 32–66% of the peak load, diagonal cracks in the clear shear span, in the main strut direction, and at the DB deep beam mid-height were noticed. In the next stages of loading, diagonal cracks propagated rapidly toward the cylindrical support and the loaded point outside edge. Their widths increased in the centre of the shear span, whereas diagonal cracks developed across the natural shear splitting line. Moreover, other diagonal cracks, parallel to the first crack, and minor vertical flexural cracks were observed in the traditionally reinforced deep beam without fibres. The crack pattern in comparison with the principal strains of the DB deep beam without fibres at peak load is shown in Figure 9.

**Figure 10:**
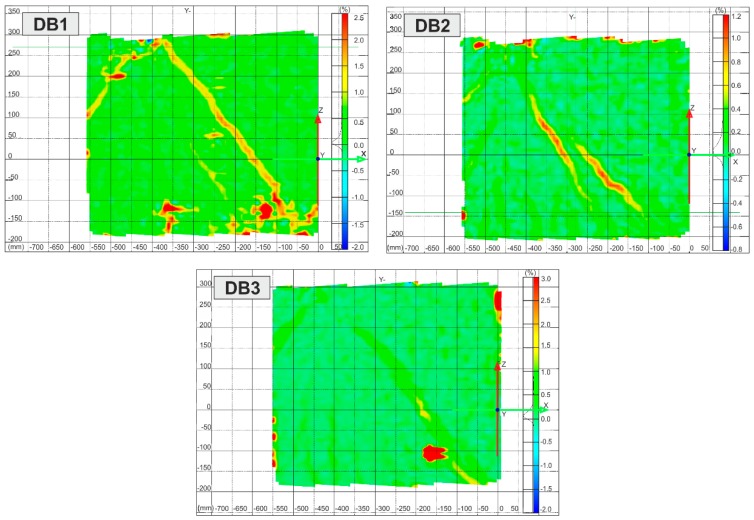
Contours of principal strains at load of 520 kN.

On the other hand, the cracks propagated and widened more slowly in the DB deep beams with fibres. The principal strain images at the load of 520 kN in Figure 10 illustrate the positive impact of hybrid fibre reinforcement on crack development.

**Figure 11:**
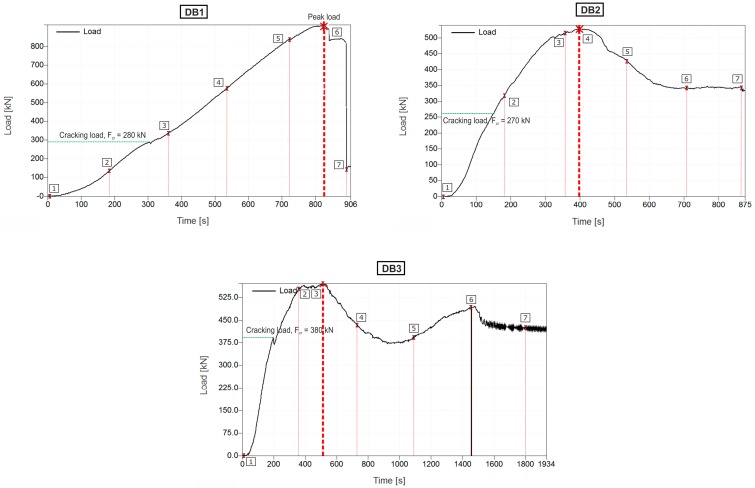
DB load–time curves.

**Figure 12:**
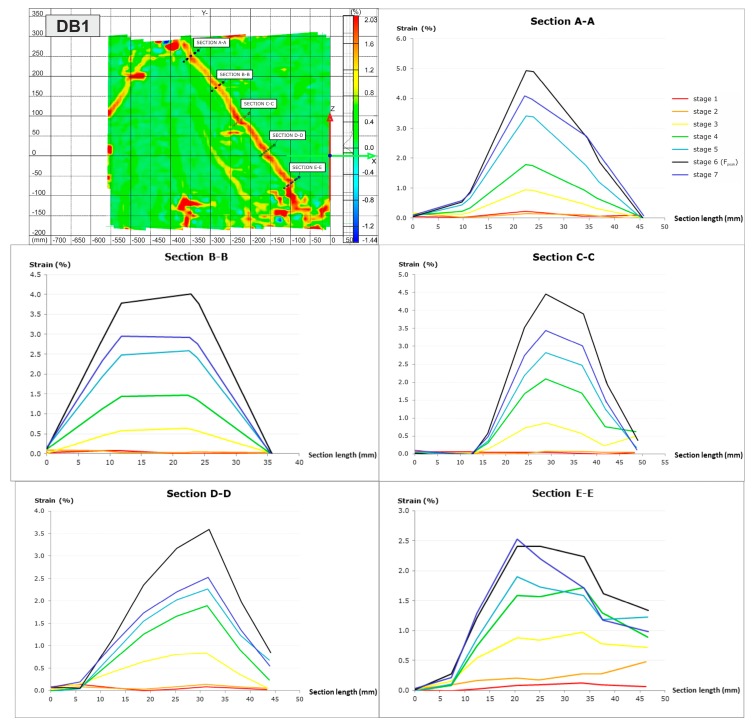

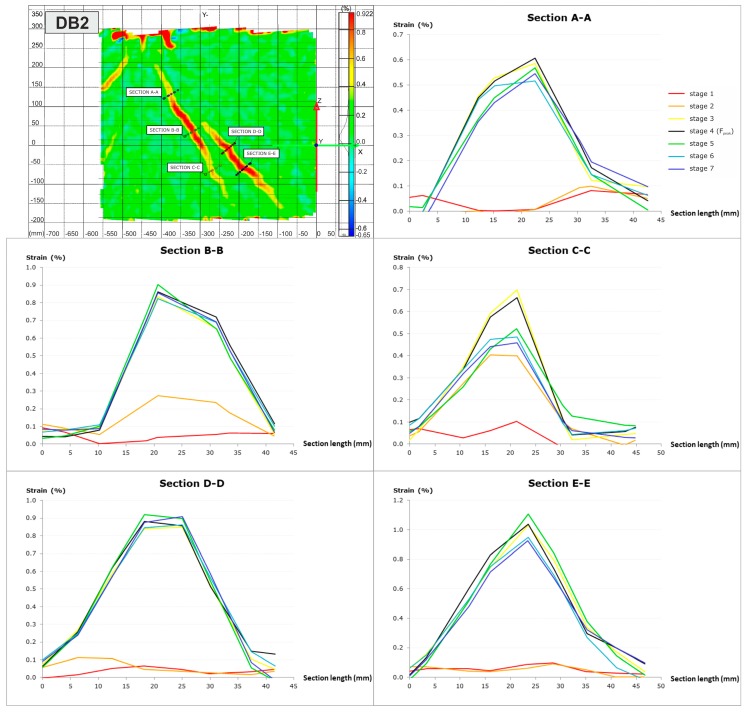

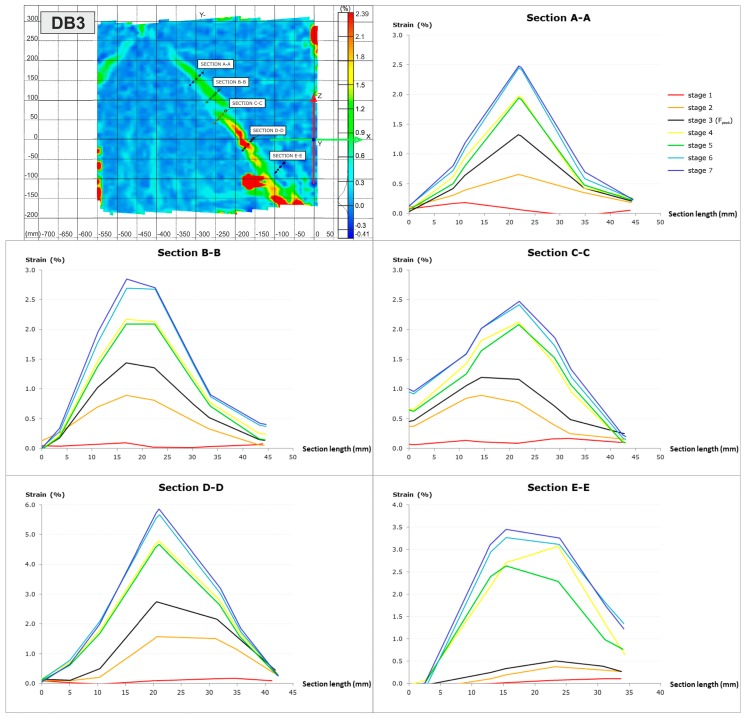
Strains in sections crossing diagonal crack of concrete deep beam (DB1) and hybrid fibre-reinforced concrete deep beams (DB2, DB3).

Figure 11 depicts the load-time curves for DB deep beams. The cracking loads, peak loads, and the points corresponding to the loading stages in which the strains in the A-A–E-E sections crossing the main diagonal crack have been marked on the curves. These strains of the concrete and hybrid FRC in the deep beams DB, composed with the location of sections are shown in Figure 12. Based on the non-contact measurements, the essential alterations in the strains of the hybrid fibre-reinforced concrete and concrete can be noticed.

The largest strains in the DB1 deep beam arise in the peak load stage and range from 2.5% in the E-E cross-section located nearby to the support in 1/5 of the deep beam height to 5% in A-A section located near the load application area at 9/10 deep beam height. As can be seen, an even increase in strain occurred during the load. In the DB2 with 1.25% fibres, two main diagonal cracks were formed. The maximum strains were almost always created at the peak load stage and ranged from 0.6% (in 2/3 of the deep beam in section A-A) to 1.1% (in 1/4 of the deep beam height in section E-E next to the support). In turn, the highest total strain in the two C-C and E-E sections located at the same height was 1.65% at the peak load and it was 36% lower than the strain of the DB1 deep beam reinforced with steel bars at the similar loading stage. The DB3 deep beam also has two main diagonal cracks with a smaller spacing than in the deep beam DB2. The highest strains occurred always in the last stages of loading and exceeded the strain at peak load twice, which were equal to 1.25% and 2.8% in the A-A cross sections (in 2/3 height) and D-D cross sections (in 2/5 height of the deep beam), respectively. Comparing the strain of both deep beams with fibres during loading history, it can be seen that a uniform strain gain similar to a traditional reinforced deep beam occurs only for the DB3 deep beam with the highest fibre volume content of 2.5%. This may indicate the beneficial effect of PP fibres in crack bridging in the early stages of loading only at a content of 0.5% and a smooth absorption of stresses by steel fibres in further stages of loading.

**Figure 13:**
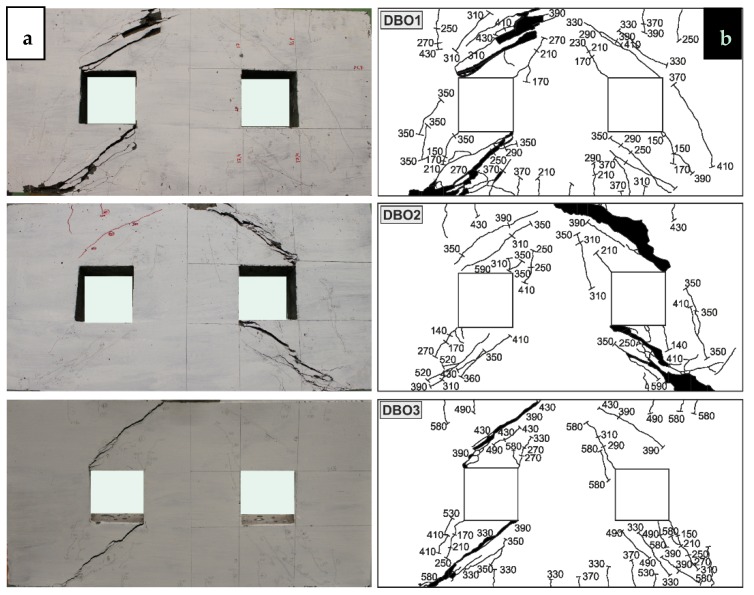
(**a**) Crack patterns and (**b**) crack propagation in DBO.

The crack patterns of the test specimens with openings are shown in Figure 13. At the opposite corners of the opening, diagonal cracks in the DBO beams first appeared, in the direction of the support and loading points. The cracking load fell within the range of 23–25% of the peak load. With each increase in load, crack expansion and propagation occurred both in the directions of the loading and support points. Minor flexural cracks occurred in the DBO1 and DBO3 deep beams at a load of approximately 50% of the peak load. A significant increase in the diagonal crack width was recorded at loads within 83–89% of the peak load. At the support crushed concrete was observed. One is able to notice that the opening significantly affects the cracking load. The presence of openings in the deep beam reduced the cracking load. The cracking load of the specimens with openings was on average 2.3 times lower than the specimens without them. This is due to the fact that the opening interrupts the natural load path.

**Figure 14:**
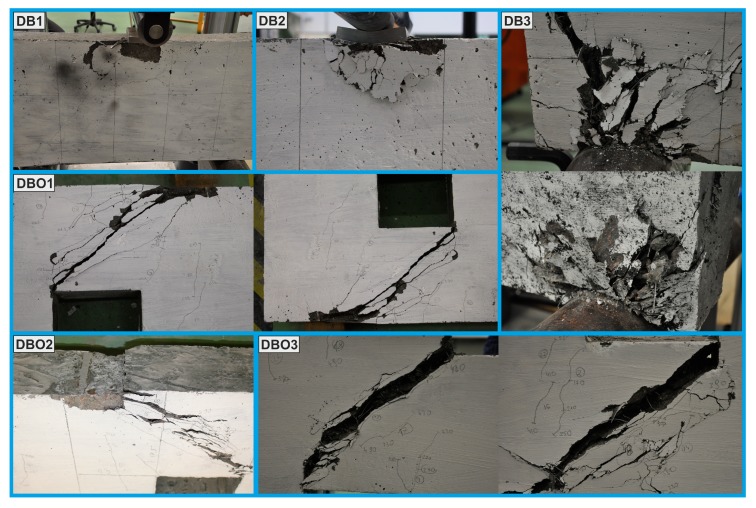
Failure modes of deep beams.

The failure modes were identified as shear-compression failure near the loading point and at the cylindrical support after the formation of several parallel diagonal cracks and propagation toward the loading points or supports (DB1, DB2, DB3); shear-compression failure near the loading area and near the support after the formation of diagonal cracks in the chords below and above of the openings (DBO1); strut crushing failure owing to the fact that above and below the openings parallel diagonal cracks appeared in the chords (DBO2); splitting the beam into two segments bridged by the fibres after the appearance of diagonal cracks in the chords below and above of the openings (DBO3), see Figure 14. The failure of the deep beams without fibres was brittle and rapid, while, all the deep beams containing the steel and polypropylene fibre reinforcement exhibited considerable softening behaviour after peak load. However, the FRC beams with openings (DBO2, DBO3) were able to resist higher shear stress than RC beam with openings (DB1). In the deep beams which had no fibres (DB1, DBO1), some spalling occurred at the failure load. 

The addition of polypropylene and steel fibres prevented spalling at failure but introducing 1%, 2% content by volume hooked-end steel fibres 50 mm in length, 1 mm in diameter, and 0.25%, 0.5% straight polypropylene fibres 12 mm in length, 25 µm in diameter into deep beams DB2 and DB3, respectively, resulted in a 38% drop in the peak load in comparison with the beam without fibres—DB1. The reason for the concrete crushing at the loading point (DB2) and at supports (DB3) can be inhomogeneous dispersion of the fibres in the deep beams. It can be seen that a high fibre content (especially polypropylene fibre) and low water/binder ratio caused poor workability and incomplete consolidation in these cases. For this reason, in the beams with openings DBO2, DBO3, the fibre content was reduced, which has a beneficial effect on the fibre density and increased the shear resistance. It was concluded that the high efficiency of hybrid fibre reinforcement is mainly dependent on the fibre volume content, its uniform distribution and the perpendicular orientation of the fibres to the direction of the cracks formed in the deep beams.

**Figure 15:**
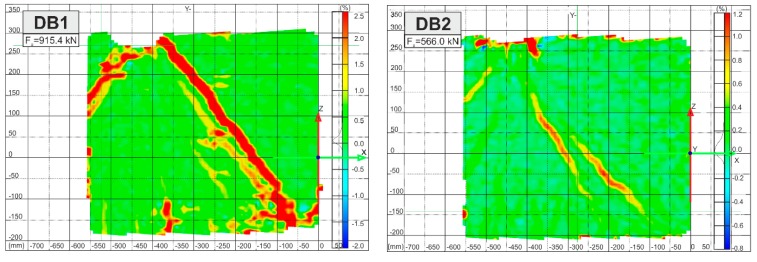

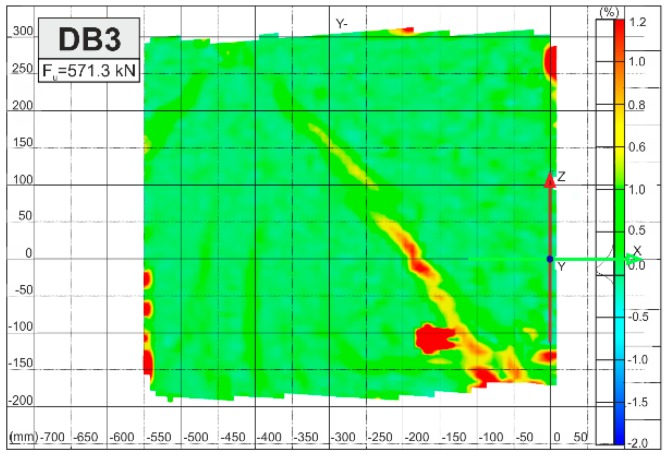
Contours of principal strains at peak load.

The impact of hybrid fibre reinforcement on the principal strains at the peak load is shown in Figure 15. It was observed that as the fibre volume content increases, the number of cracks decreases significantly. From a practical point of view, these findings support the concept that hybrid fibres could be introduced to deep beams at a 1.5% content by volume of steel fibre and 0.1% polypropylene fibre. The investigation shows that the addition of steel and polypropylene fibres can replace the web mesh in high-performance concrete deep beams.

**Figure 16:**
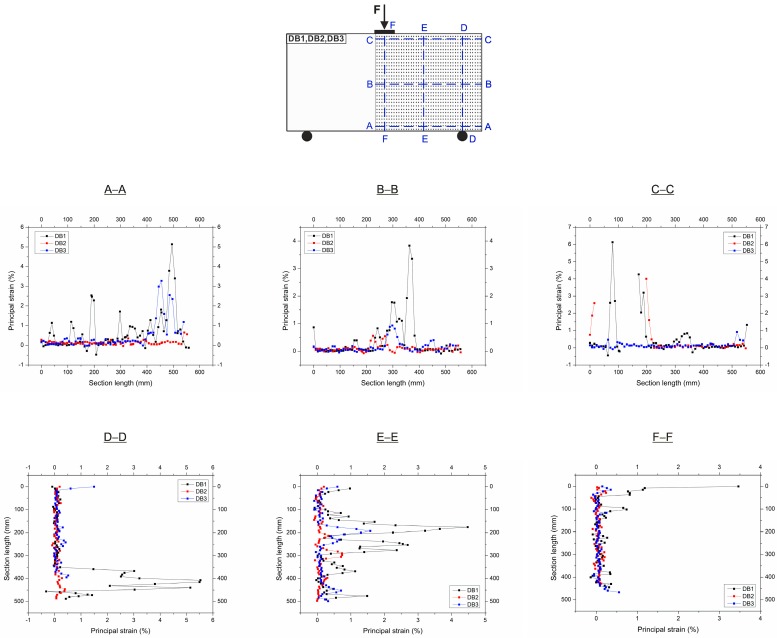
Principal strains of RC and fibre-reinforced concrete (FRC) deep beams at peak load.

The principal strains in the RC and hybrid FRC deep beams DB with the location of horizontal and vertical cross-sections are shown in Figure 16. The principal strain curves were made in horizontal sections A-A, B-B and C-C and vertical sections D-D, E-E and F-F which were localized in the shear span of deep beams DB1, DB2, DB3.

It can be noticed the essential differences in the strain distributions of HPC and hybrid fibre reinforced HPC. The highest strains in the DB1, DB2 and DB3 deep beams at the peak load near the bottom edge of the elements at the support zone (cross-section A-A) are 5.2%, 0.7% and 3.3%, respectively. The maximum strains in the middle of the shear span at half height (B-B) decreased slightly, and near the top edge (C-C) increased in the DB1 and DB2 deep beams, and decreased for the DB3 element with the highest fibre content. Similar tendencies were observed in vertical cross-sections (D-D, E-E, F-F). In general, the most favourable deformation distribution in all cross-sections was observed for the DB3 deep beam. This indicates a very good interaction between hybrid fibres, HPC, and steel reinforcement in reducing deformation.

### 3.3. Parameters of Deep Beams

**Figure 17:**
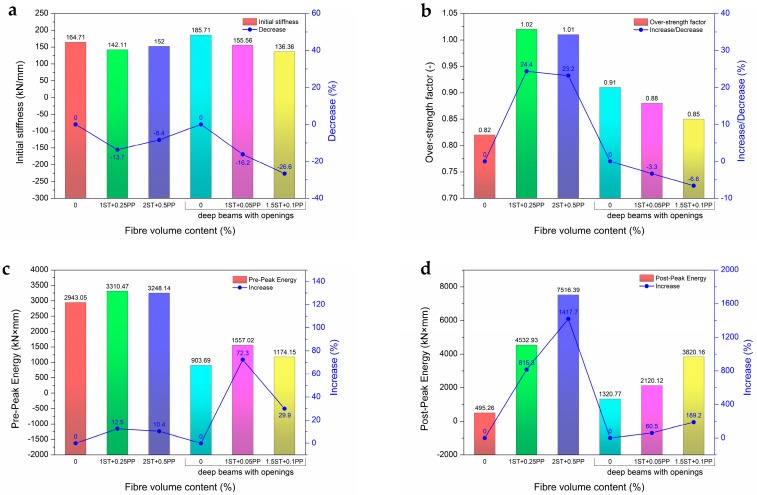

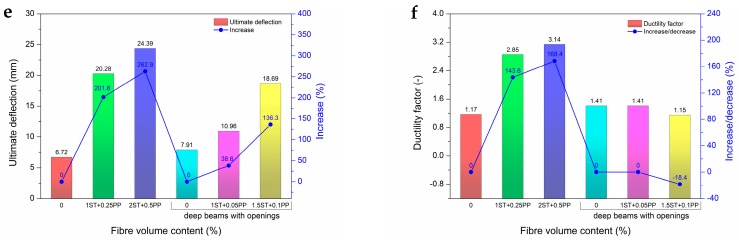
Comparison of deep beam (**a**) initial stiffness; (**b**) over-strength factor; (**c**) pre-peak energy; (**d**) post-peak energy; (**e**) ultimate deflection and (**f**) ductility factor.

The elastic and inelastic parameters of the deep beams are shown in Table 5 and Figure 17. The initial stiffness is computed as the first cracking load divided by the corresponding deflection. The over-strength is specified as the reserve strength of the structure member and capacity to dissipate energy. It depends on different factors in which the most important of them are the structural element geometry, strain hardening, fibre content and ductility. The over-strength factor is calculated as the ratio of failure load to the yield load of the member [63]. The ductility factor is defined as the failure structural deflection to the yield strength deflection [62]. Figure 8b depicts definition of these factors. The pre- and post-peak energies of deep beams are assessed by the integration of the area under the load-deflection curve is divided into two regions [64].

**Figure 18:**
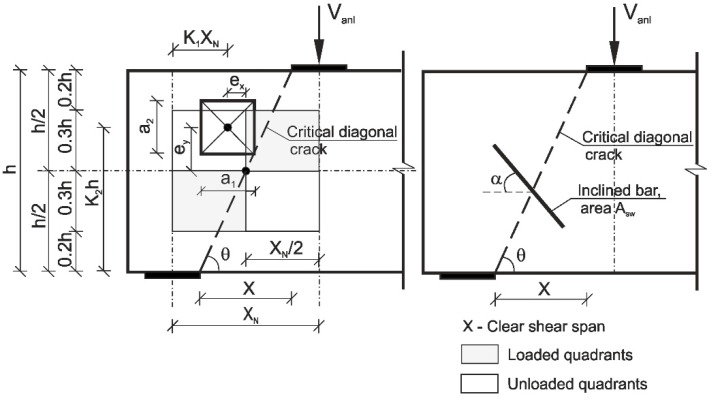
Idealization of deep beam with openings [30,33].

The initial stiffness of the fibre deep beams shows lower values in comparison with that of reinforced concrete deep beams (Figure 17a). The decrease in the initial stiffness of the fibre deep beams is affected by the increase in cracking deflection at corresponding load, which are effects of the different arrangement of reinforcement associated with a fibre volume content as well as the lack of web meshes. The FRC deep beams without openings have higher over-strength factor values in comparison with RC deep beam DB1, Figure 17b. This shows the dominant action of web meshes in the first stage of strain hardening. The pre- and post-peak energies values are calculated and shown in Figure 17c,d for comparing the influence of fibre content on increasing the energy absorption capacity of deep beams with/without openings. As can be seen, the lower increases in values of pre- and post-peak energies for FRC belong to deep beams DBO2 and DBO3 with a square opening in the shear spans. The ultimate inelastic deflection and ductility factor of the deep beams are plotted in Figure 18e,f. Deep beams DB3 and DBO3 with the highest hybrid fibre content, 2.5% and 1.6%, obtained 3.6-fold and 2.4-fold increase in the ultimate deflection, respectively. In consequence, the ductility factors in general indicate the positive impact of hybrid fibres on increasing the ductility. A decrease of 18% in the ductility factor is noted for deep beam DBO3 with openings.

## 4. Analysis of Failure Modes

A method to analyse the shear strength of traditional reinforced high performance concrete deep beams with and without openings (DB1, DBO1) and steel-polypropylene fibre-reinforced high performance concrete deep beams with and without openings (DB2, DB3, DBO2, DBO3) is examined in this section. The ultimate shear capacity was calculated on the basis of Equations (1)–(8) for reinforced concrete deep beams and its modified form taking into account the presence of steel fibres [30,40]. The experiments on the deep beams with openings proved that failure can occurred by a creation of two independent diagonal cracks in the chords above and below of the opening [33]. Figure 18 presents the idealization of reinforced concrete deep beams with openings.

The reinforced concrete shear strength of deep beams with/without openings and with/without fibres was calculated using the following equation

(1) $$V_{anl}=V_{s}+V_{c}$$

where

$V_{s}$: contribution of the steel bars on shear strength by dowel action,

$V_{c}$: contribution of concrete or fibre-reinforced concrete on shear strength.

Equations (2)–(8), taken from [30,33], were applied to estimate the contribution of steel bars and fibre reinforced concrete.

(2) $$V_{s}=\Psi_{s}\sum A_{s}f_{y}\left(\frac{\tan\theta \tan\phi -1}{\tan\theta +\tan\phi }\right)+\Psi_{sw}\sum A_{sw}f_{yw}\left[\frac{\sin\alpha \cos\theta +\cos\alpha }{\left(\frac{\tan\theta +\tan\phi }{\tan\theta \tan\phi }\right)}-\frac{\cos\alpha }{\left(\frac{\tan\theta +\tan\phi }{1-\tan\alpha \tan\theta }\right)}\right]$$

(3) $$\tan\phi =\frac{f_{c}-f_{ct}}{2\sqrt{f_{c}f_{ct}}}$$

(4) $$V_{c}=f_{1}f_{2}f_{3}\frac{cbh}{\sin\theta \cos\theta \left(\tan\theta +\tan\phi \right)}$$

(5) $$c=\frac{\sqrt{f_{c}f_{ct}}}{2}$$

(6) $$f_{1}=\{\begin{array}{ccc} 1-\frac{1}{3}\left(\frac{K_{1}X_{N}}{K_{2}h}\right) & \mathrm{for} & \frac{K_{1}X_{N}}{K_{2}h}\leq 1\\ \frac{2}{3} & \mathrm{for} & \frac{K_{1}X_{N}}{K_{2}h}>1 \end{array}$$

(7) $$f_{2}=\left(1-\frac{a_{1}}{2X}\right)\left(1-\frac{a_{2}}{1.2h}\right)$$

(8) $$f_{3}=\{\begin{array}{cc} \left(0.85+0.3\frac{e_{x}}{X_{N}-a_{1}})(0.85+0.3\frac{e_{y}}{0.6h-a_{2}}\right) & \text{for opening centre in unloaded quadrant}\\ \left(0.85-0.3\frac{e_{x}}{X_{N}-a_{1}})(0.85-0.3\frac{e_{y}}{0.6h-a_{2}}\right) & \text{for opening centre in loaded quadrant} \end{array}$$

where

$\Psi_{s}=$ empirical coefficient reflecting the level of stress in steel rebar,

$\Psi_{s}=0.5$ for DB deep beams with fibres,

$\Psi_{s}=0.9$ for DBO deep beams [30,33],

$\Psi_{sw}=$ empirical coefficient reflecting the location of web reinforcement,

$\Psi_{sw}=0.5$ [30],

$A_{s}=$ steel rebar area,

$A_{sw}=\;$ inclined web steel bar area (Figure 18),

$f_{y}=$ steel rebar yield point stress,

$f_{yw}=$ yield point stress of web reinforcement intercepted by the critical diagonal crack,

θ= inclination angle of the natural shear path to the horizontal,

∅=  angle of internal friction of concrete given by the Mohr–Coulomb failure criterion,

α= web bar inclination angle to the horizontal (Figure 18),

$f_{c}=$ concrete compressive strength,

$f_{ct}=$ concrete tensile splitting strength,

c= concrete cohesion given by the Mohr–Coulomb theory,

b= deep beam width,

h= deep beam height,

$f_{1}=$ coefficient defining the opening location,

$f_{2}=$ coefficient defining the size of the opening,

$f_{3}=$ coefficient reflecting the combined effect of the size of the opening and its location, can reach any value between 0.5 and 1,

$X_{N}=$ nominal shear span,

$K_{1},K_{2}=$ opening location coefficients (Figure 18),

X= clear shear span,

$a_{1},a_{2}=$ opening dimensions,

$e_{x},e_{y}=$ web opening centre eccentricities.

The experimental and theoretical results are compared in Table 6.

It was noticed that the shear strength of the fibre-reinforced deep beams (DB2, DB3) were slightly overestimated by the analytical model. For these specimens, the ratios were 0.97 and 0.96, respectively. The slight overestimate of load-carrying capacity in these cases is due to the larger variability of the results obtained for the deep beams without web reinforcement. Placing web reinforcement (in DB1) considerably reduces the variability and the predicted value appear on the safe side. Traditional reinforced concrete deep beam with openings (DBO1) was overestimated by 22% by the analytical model. This probably means that model coefficients reflected the stress in the steel rebar just prior to failure. Table 6 indicates a good correlation between predicted and experimental values for the case of fibre-reinforced deep beams with openings subjected to single-point loading, with the predicted values always being on the safe side. The test to theoretical shear strength ratios of all the specimens were between 0.82–1.19. The analytical model accurately predicts the shear strength of deep beams with and without web openings made from hybrid fibre reinforced high performance concrete, taking into consideration the complexity of the issue.

## 5. Conclusions

The following conclusions were drawn on the basis of the results obtained from the experimental investigations and theoretical analyses.

The load at the first macro-crack of the DB fibre reinforced concrete deep beams decreases with the addition of hybrid fibres in comparison to traditionally reinforced concrete deep beams. In contrast, the load at the first crack of the DBO deep beams with fibres and openings slightly increases with a greater fibre content. In comparison to the deep beams without fibres, the specimens with a mixture of steel and polypropylene fibres have narrower crack widths.

The fibre-reinforced concrete deep beam specimens with openings containing a steel fibre content from 1% to 1.5% and polypropylene fibre content from 0.05% to 0.1% exhibits a 28% greater ultimate shear strength. The strength of the deep beams increases considerably thanks to the hybrid fibres present around the openings. A reduction in shear strength of about 38% is noted for the fibre reinforced deep beams without openings with 1–2% steel fibre and 0.25–0.5% polypropylene fibre content by volume. The reason is the high content of polypropylene fibres, resulting in poor fibre dispersion in the deep beams without openings.

The failure modes are dependent on the fibre volume contents, uniform distribution and perpendicular orientation to formed cracks. The specimens with openings fail owing to the fact that several independent diagonal cracks form in the chords below and above the opening.

Values of the post-peak energy, ultimate deflection and ductility factor are highest for hybrid fibre content in a combination of 2% steel fibres and 0.5% polypropylene fibres.

Hybrid fibres added to the concrete mix are able to replace conventional steel web meshes in deep beams with and without openings.

The equations predicting the shear strength of hybrid fibre-reinforced high performance concrete deep beams with and without web openings as well as the empirical coefficients reflecting the level of stress in steel rebar and the location of web reinforcement should be verified for a higher number of representative deep beam elements.

## Figures and Tables

**Table 1:** Mixture proportions.

Material	Symbol Unit	DB1	DB2	DB3	DBO1	DBO2	DBO3
Cement CEM I 52.5R	C (kg/m^3^)	596	596	596	596	596	596
Silica fume	M (kg/m^3^)	149	149	149	59.6	59.6	59.6
Granodiorite 2/8 mm	A (kg/m^3^)	990	990	990	990	990	990
Quartz sand 0.05/2 mm	S (kg/m^3^)	500	500	500	500	500	500
Superplasticiser	SP (l/m^3^)	39	39	39	20	20	20
Water	W (l/m^3^)	139	139	139	177	177	177
Steel fibre	ST (kg/m^3^)	-	78	156	-	78	117
	V_ST_ (%)	-	1	2	-	1	1.5
Polypropylene fibre	PP (kg/m^3^)	-	2.3	4.5	-	0.5	1
	V_PP_ (%)	-	0.25	0.5	-	0.05	0.1

**Table 2:** Mechanical properties of concrete and fibre reinforced concrete.

Concrete Properties	Unit	DB1	DB2	DB3	DBO1	DBO2	DBO3
Compressive strength	MPa	114.2	88.1	84.7	113.0	113.6	112.4
Splitting tensile strength		5.3	6.9	7.4	6.2	10.2	10.4
Flexural tensile strength		6.9	8.9	8.3	7.4	8.7	9.1
Modulus of elasticity		38741	39598	39370	38251	38964	39131

**Table 3:** Mechanical properties of the reinforcement.

Reinforcement	Diameter (mm)	Yield Strength (MPa)	Tensile Strength (MPa)	Ultimate Elongation (%)	Modulus of Elasticity (GPa)
Tension bar	22	457	644	10.5	203
Compression and diagonal bar	12	456	642	9.7	199
Web mesh	8	447	640	9.6	196
Stirrup	6	302	454	8.5	193

**Table 4:** Load-deflection response results of deep beams at cracking, yielding, peak and failure loads.

Deep Beam Notation	Fibre Volume Content (%)	Cracking		Yielding		Peak		Failure	
		Load F_cr_ (kN)	Deflection α_cr_ (mm)	Load F_y_ (kN)	Deflection α_y_ (mm)	Load F_peak_ (kN)	Deflection α_peak_ (mm)	Load F_fail_ (kN)	Deflection α_fail_ (mm)
DB1	-	280	1.7	891	5.7	915	6.1	732	6.7
DB2	1 ST + 0.25 PP	270	1.9	442	3.9	566	8.2	453	11.1
DB3	2 ST + 0.5 PP	380	2.5	453	3.5	571	7.8	457	11.0
DBO1	-	130	0.7	412	2.9	466	3.4	373	4.1
DBO2	1 ST + 0.05 PP	140	0.9	546	3.7	601	4.5	481	5.2
DBO3	1.5 ST + 0.1 PP	150	1.1	560	3.4	598	3.8	479	3.9

**Table 5:** Deep beam parameters.

Deep Beam Notation	Fibre Volume Content (%)	Initial Stiffness (kN/mm)	Over-Strength Factor (–)	Pre-Peak Energy (kN × mm)	Post-Peak Energy (kN × mm)	Ductility Factor (–)
DB1	-	165	0.82	2943	495	1.17
DB2	1 ST + 0.25 PP	142	1.02	3310	4533	2.85
DB3	2 ST + 0.5 PP	152	1.01	3248	7516	3.14
DBO1	-	186	0.91	904	1321	1.41
DBO2	1 ST + 0.05 PP	156	0.88	1557	2120	1.41
DBO3	1.5 ST + 0.1 PP	136	0.85	1174	3820	1.15

**Table 6:** Experimental and theoretical results for deep beams.

Deep Beam	Experimental *V*_exp_ (kN)	Analytical			Ratio *V*_exp_/*V*_anl_
		*V*_s_ (kN)	*V*_c_ (kN)	*V*_anl_ (kN)	
DB1	915.4	549.2	359.3	908.5	1.01
DB2	566.0	158.8	426.4	585.2	0.97
DB3	571.3	146.4	447.9	594.3	0.96
DBO1	466.2	140.9	426.5	567.4	0.82
DBO2	600.8	295.2	211.9	507.1	1.18
DBO3	598.3	289.7	214.8	504.5	1.19
